# Branched chain amino acids metabolism in heart failure

**DOI:** 10.3389/fnut.2023.1279066

**Published:** 2023-11-20

**Authors:** Chenshan Gao, Lei Hou

**Affiliations:** ^1^Collaborative Innovation Centre of Regenerative Medicine and Medical BioResource Development and Application Co-Constructed by the Province and Ministry, Guangxi Medical University, Nanning, China; ^2^Department of Cardiology, Shanghai Songjiang District Central Hospital, Shanghai Jiao Tong University School of Medicine, Shanghai, China

**Keywords:** branched-chain amino acids, heart failure, metabolic dysregulation, HFrEF – heart failure with reduced ejection fraction, HFpEF – heart failure with preserved ejection fraction

## Abstract

As a terminal stage of various cardiovascular diseases, heart failure is of great concern due to its high mortality rate and limited treatment options. Researchers are currently focusing their efforts on investigating the metabolism of carbohydrates, fatty acids, and amino acids to enhance the prognosis of cardiovascular diseases. Simultaneously, branched-chain amino acids (BCAAs), including leucine, isoleucine, and valine, play significant roles in blood glucose regulation, protein synthesis, and insulin sensitivity. However, disrupted BCAAs metabolism has been associated with conditions such as hypertension, obesity, and atherosclerosis. This article explores intricate metabolic pathways, unveiling the connection between disrupted BCAAs metabolism and the progression of heart failure. Furthermore, the article discusses therapeutic strategies, assesses the impact of BCAAs on cardiac dysfunction, and examines the potential of modulating BCAAs metabolism as a treatment for heart failure. BCAAs and their metabolites are also considered as biomarkers for evaluating cardiac metabolic risk. In conclusion, this article elucidates the multifaceted roles of BCAAs in heart failure and cardiovascular health, providing guidance for future research and intervention measures.

## Introduction

Leucine (Leu), isoleucine (Ile), and valine (Val) constitute essential branched-chain amino acids (BCAAs), acquired solely through dietary consumption (1–5). Serving as both signaling molecules and regulators, BCAAs play a pivotal role in diverse physiological processes. These processes encompass the maintenance of blood glucose balance, facilitation of protein synthesis, modulation of insulin resistance, and regulation of pathways associated with nutrient sensitivity (2–9). These roles highlight BCAAs’ multifaceted impact on various physiological functions. As advancements in amino acid research shed light on BCAAs’ roles, some studies propose that beyond being dietary nutrients, BCAAs may also contribute to regulating specific diseases. Notably, several studies have revealed a significant association between BCAAs and heart failure (HF), encompassing aspects of its progression, severity, and prognosis. However, further investigation is necessary to precisely determine the functional role of BCAAs in HF. This paper aims to fill this gap by providing a concise overview of the current research progress on the physiological and pathophysiological processes related to BCAAs in HF. Additionally, this paper explores the potential application, feasibility, and significance of harnessing BCAAs and their metabolism in the treatment of HF. Through this comprehensive exploration, we aim to contribute to a deeper understanding of BCAAs’ therapeutic potential in the context of heart failure.

### Metabolic pathways and regulation of branched-chain amino acids

Tracer studies in mice have demonstrated that the oxidation of BCAAs primarily occurs in skeletal muscles, brown adipose tissue, liver, kidneys, and heart. Notably, the liver, pancreas, skeletal muscles, kidneys, and brown adipose tissue also play essential roles in BCAA protein synthesis (10). The initial metabolism of BCAAs takes place in extrahepatic tissues through enzymatic reactions involving isoleucine, leucine, and valine, with these metabolic pathways being conserved among eukaryotes (11).

The initiation of branched-chain amino acids (BCAAs) metabolism involves a reversible transamination process catalyzed by the enzyme BCAT2 within the mitochondria, resulting in the formation of branched-chain α-keto acids (BCαKAs). These BCαKAs subsequently undergo an irreversible decarboxylation process mediated by the enzyme complex BCKDH. The activity of the BCKDH complex is tightly regulated through mechanisms of phosphorylation and dephosphorylation, as demonstrated by various studies (5, 7, 9–12). Branched-chain α-keto acid dehydrogenase kinase (BCKDK) phosphorylates and inhibits branched-chain α-keto acid dehydrogenase, while its activation is facilitated by dephosphorylation mediated by the phosphatase PPM1K, also known as PP2Cm (13). Notably, exposure to BCαKAs, particularly α-KIC, suppresses BCKDK, promoting the kinase’s oxidative metabolism and resulting in elevated BCαKAs levels. The subsequent reactions involving these BCαKAs contribute to the synthesis of end metabolites such as acetyl-CoA and succinyl-CoA, which actively participate in the oxidative steps of the tricarboxylic acid (TCA) cycle (3–5, 11).

Under normal physiological conditions, the intricate balance between the synthesis and consumption of amino acids directly influences protein synthesis. This balance extends to the dynamic interplay between branched-chain amino acids (BCAAs) and branched-chain α-keto acids (BCαKAs), primarily governed by the activity of the pivotal enzyme, BCαKAs dehydrogenase, within the oxidative metabolism pathway of BCAAs (3, 5). Therefore, the precise regulation of BCαKAs dehydrogenase expression and activity emerges as a critical determinant for preserving the delicate equilibrium of BCAAs within the body’s cycling pool (14, 15).

The oxidative metabolism of BCAAs can lead to two outcomes: termination in the tricarboxylic acid cycle or the generation of intermediates with paracrine signaling activity, such as 3-hydroxyisobutyric acid (3-HIB). This process also produces monomethyl-branched chain fatty acids (mmBCFAs) (16, 17). Turning our focus to leucine, an essential BCAA, it activates mTOR in the heart, blocking ULK1 and inhibiting autophagy. Additionally, leucine promotes insulin resistance by phosphorylating IRS-1 via S6K and stimulates protein synthesis through the phosphorylation of 4E-BP1 (18). Furthermore, branched-chain α-keto acids (BCαKAs) enhance 4E-BP1 phosphorylation and activate the MEK–ERK protein kinase pathway, thus promoting protein synthesis (7). Notably, exposure to BCAAs negatively impacts cardiac mitochondrial complex activity, leading to the generation of reactive oxygen species and oxidative stress (3).

### Impact of BCAAs on heart failure

Heart failure (HF) represents the clinical consequence of diverse cardiovascular diseases (CVDs), characterized by compromised heart structure or function that disrupts the coordinated ventricular contraction and filling processes. This disruption manifests as a complex array of intricate symptoms and signs. Categorically, HF is delineated by left ventricular ejection fraction (LVEF), wherein LVEF ≤40% defines HF with reduced ejection fraction (HFrEF), while LVEF ≥50% signifies HF with preserved ejection fraction (HFpEF). A compelling cross-sectional study conducted by Ruiz-Canela et al. substantiated the nexus between branched-chain amino acids (BCAAs) and escalated CVD risk (19). Remarkably, valine and leucine emerged as autonomous biomarkers, signifying augmented cardiovascular and metabolic jeopardy, independently of body mass index (BMI). Previous studies reporting on the association between BCAAs and HF have been conducted in several cohorts (Table 1).

**Table 1:** Human epidemiological studies showing associations between BCAAs and heart failure.

Endpoint	Population	Findings	Refs.
Heart failure with reduced ejection fraction	HF patients	Elevated plasma BCAAs levels in HF patients	(3, 20, 21)
	DCM patients	Cardiac BCAA levels were dramatically elevated in left ventricular samples of patients with DCM	(11, 18)
Heart failure with preserved ejection fraction	T2DM patients	BCAAs in the fasting serum of type 2 diabetes mellitus (T2DM) patients have autonomously correlated with the emergence of incident HF, higher BCAA levels in people with and prior to development of type 2 diabetes	(22, 23)
	Obesity	The very fabric of obesity and insulin resistance orchestrates the repression of genes integral to BCAAs degradation, elevations in circulating BCAAs have gained attention as potential contributors to the development of insulin resistance and diabetes	(24)
	High cardiovascular risk population	Higher concentrations of baseline BCAAs were associated with increased risk of CVD, especially in a high cardiovascular risk population	(19)
	The Japanese population without diabetes mellitus.	In both sexes, the levels of individual BCAAs and the total BCAA levels correlated positively with triglyceride levels and negatively with high-density lipoprotein cholesterol levels	(25)
	HF patients	Elevated plasma BCAA levels have been distinctly discerned in HF patients with impaired systolic function when juxtaposed with those exhibiting sustained diastolic function and individuals devoid of HF	(26)

### Heart failure with reduced ejection fraction

A slew of investigations has concretely established the correlation between augmented circulating plasma BCAAs and subsequent adversities in HF patients (3, 20, 21). This propensity for elevated plasma BCAAs levels has been mirrored in animal models of HF, including myocardial infarction-induced HF in rats, positing an intricate connection between BCAAs and cardiac malaise (27). Studies have revealed that elevated levels of BCAAs impede glucose metabolism and increase the vulnerability of the heart to ischemic injury in a mouse model with impaired BCAAs metabolism (19). Advanced scrutiny has divulged the deranged BCAAs metabolism intrinsic to cardiac tissues, marked by dwindling BCAAs breakdown enzymes and an accrual of BCAAs within the cardiac milieu (28). Intriguingly, a study involving pressure-induced HF in mice spotlighted heightened levels of BCαKAs, enzymes pivotal in BCAAs degradation, corroborating the conjecture of distorted BCAAs metabolism in cardiac dysfunction (29).

A groundbreaking murine HF model, induced via oxidative stress, laid bare that compromised expression of BCAAs catabolic enzymes instigates elevated BCAAs levels intrinsically within the cardiac framework, while their circulatory counterparts remain unperturbed (30). These findings accentuate that impeded BCAAs metabolism and heightened intracardiac BCAAs levels typify prevailing hallmarks of cardiac dysfunction in HF. Moreover, HF patients are characterized by escalated circulating BCAAs levels. This is coupled with the downregulation of genes governing BCAAs breakdown in HF, which prompts the accumulation of BCAAs and their metabolic derivatives, BCαKAs, within cardiac tissues. Another murine model, simulating pressure overload-induced HF, unmasked malfunctions in BCAAs degradation, oxidative stress, and metabolic derangement. This chronic BCAAs buildup in metabolically compromised mice potently retards glucose metabolism and amplifies vulnerability to ischemia–reperfusion injury. Notably, patients afflicted with dilated cardiomyopathy (DCM)-associated HF evinced escalated cardiac BCAAs levels, underscoring the pivotal role of impaired BCAAs breakdown in failing cardiac structures (11).

The inhibition of BCAAs breakdown in HF is intimately associated with the activation of the TAK1/P38MAPK signaling cascade, triggering a surge in cardiac BCAAs levels (18). Profound research has unequivocally established the efficacy of circulating BCAAs levels in distinguishing chronic HF model rats from sham-operated counterparts, thereby delineating the impaired BCAAs metabolic pathways characteristic of deteriorating cardiac function (27). This inhibition is concurrently accompanied by a diminution in PP2Cm expression (13). Elevated BCαKAs levels directly impede mitochondrial respiration, thereby fomenting oxidative stress and precipitating HF under oxidative duress and mechanical overloads, as illustrated in PP2Cm-knockout murine models (29).

### Heart failure with preserved ejection fraction

Remarkably, BCAAs in the fasting serum of type 2 diabetes mellitus (T2DM) patients have autonomously correlated with the emergence of incident HF (22). Elevated plasma BCAA levels have been distinctly discerned in HF patients with impaired systolic function when juxtaposed with those exhibiting sustained diastolic function and individuals devoid of HF (26). Surprisingly, studies have unveiled augmented BCAAs oxidation within the heart during HF, defying expectations, and the abatement of plasma and cardiac BCAAs levels did not confer appreciable protective outcomes (11). Conversely, the emergence of evidence hints at the potential of activating BCAAs catabolism in mitigating blood pressure, thereby hinting at a potential modality for cardiac safeguarding. Mendelian randomization studies have further underscored the profound impact of regulating BCAAs catabolism on human blood pressure dynamics (11). As such, targeting BCAAs α-keto dehydrogenase kinase emerges as a prospective therapeutic strategy for HF amelioration.

Metabolomic profiling studies have unequivocally unveiled the promise of BCAAs and their associated metabolites as potent biomarkers for refined cardiac metabolic risk assessment. In the context of insulin resistance, plasma BCAAs levels exhibit a proclivity for elevation, thereby magnifying the nexus between BCAAs and metabolic aberrations (31–33). An intriguing amalgamation known as the diabetes risk index (DRI), which interweaves BCAAs and LP-IR, has duly emerged as a robust biomarker predictive of type 2 diabetes mellitus (T2DM) and newly diagnosed hypertension, irrespective of traditional clinical risk factors (34, 35). The tapestry of epidemiological and experimental evidence further bolsters the observed correlation between BCAAs levels and insulin resistance (23). Moreover, elevated plasma BCAAs levels have been discerned in metabolically infirm obese individuals, when juxtaposed with their metabolically resilient obese counterparts and individuals boasting normal BMI. The very fabric of obesity and insulin resistance orchestrates the repression of genes integral to BCAAs degradation (24). The synergy between escalated BCAAs levels and dyslipidemia and metabolic maladies poignantly underscores the pivotal role of BCAAs metabolism in orchestrating metabolic equilibrium (25). Lifestyle determinants such as elevated BMI, sedentary habits, and alcohol consumption have also found their cadence in the elevation of BCAAs levels (36). Paradoxically, dietary BCAAs intake seems to cast a shadow of insignificance on the primacy of elevated plasma levels (36).

The distorted BCAAs metabolism and resulting accumulation of BCAAs and their metabolites are recurring themes in HF, surpassing ejection fraction limits. Understanding their mechanisms and implications can unveil potential HF treatment targets. Revealing BCAAs’ role in HF and metabolic changes can lead to valuable insights, fostering potent interventions. Exploring BCAAs α-ketodehydrogenase kinase and regulating catabolism offers innovative HF treatments. Leveraging BCAAs and associated metabolites as key factors in cardiac risk assessment enhances diagnostic precision and prognosis. A comprehensive grasp of BCAAs metabolism and its implications in HF and metabolic discord forms the foundation for innovative interventions and management.

### Targeting BCAAs metabolism as a potential therapeutic approach for HF

Approximately 20% of HF patients experience muscle wasting and cachexia. Research indicates that supplementation with BCAAs can improve various aspects of the condition, including the 6 min walk test distance, muscle mass, and overall quality of life (37). Furthermore, an investigation by Uchino et al. found that BCAAs supplementation in hospitalized HF patients with hypoalbuminemia resulted in a reduction in the cardiothoracic ratio and an increase in serum albumin levels, as compared to the control group (38). However, it is crucial to consider that excessive BCAAs supplementation may impose additional strain on the already compromised heart, potentially exacerbating the clinical course of the disease. Therefore, a delicate balance in BCAAs supplementation is essential for optimizing therapeutic outcomes. Different strategies have been investigated to improve cardiac BCAAs oxidation and decrease plasma levels of BCAAs in preclinical models of heart HF due impaired cardiac BCAAs oxidation on cardiac energy metabolism, function, and structure (2, 18). For instance, a study involving pressure-overloaded HF mice demonstrated that the inhibition of amino acid dehydrogenase kinase using BT2, an amino acid dehydrogenase kinase inhibitor, enhanced BCAAs metabolism, facilitated the utilization of fatty acids by the heart, and ultimately improved systolic and diastolic functions (39). These findings highlight the potential of precisely targeting the BCAAs metabolic pathway as a promising treatment approach for HF within the cardiovascular field.

BCAAs play a critical role in protein synthesis, and their metabolism is intricately linked to overall health and disease. The PI3K-AKT-mTOR pathway serves as a pivotal signaling pathway connecting these physiological states. As BCAAs and their metabolites emerge as potential biomarkers and contributors to HF, an in-depth understanding of their regulatory roles can unveil new avenues for therapeutic interventions. Efficient modulation of BCAAs metabolism can potentially alleviate HF by restoring cardiac energy balance and function. Moreover, evidence suggests that BCAAs and their derivatives also hold promise as biomarkers for assessing cardiovascular risk, including coronary artery disease and HF. However, further research is warranted to elucidate the effectiveness of plasma detection of BCAAs and their metabolites in predicting a broader spectrum of cardiovascular diseases, such as atherosclerosis, hypertension, and arrhythmias. In conclusion, targeting the BCAAs metabolic pathway presents a promising therapeutic approach for HF within the cardiovascular field. A comprehensive understanding of the roles played by BCAAs and their metabolites in the context of HF can inform the development of effective treatment strategies and the identification of novel biomarkers for a range of cardiovascular diseases. By bridging the gap between metabolic dysregulation and cardiovascular health, this research paves the way for innovative interventions and improved patient outcomes.

## Conclusion

Branched-chain amino acids (BCAAs) – leucine, isoleucine, and valine – are crucial dietary nutrients with diverse roles in physiology. They regulate blood glucose, promote protein synthesis, and affect insulin resistance. Recent research highlights their connection to heart failure (HF), with elevated BCAAs levels influencing disease progression. In HF, impaired BCAAs metabolism, characterized by reduced breakdown and cardiac tissue accumulation, plays a key role. Understanding the role of enzymes like BCKDH and BCAAs dehydrogenase kinase in BCAAs metabolism holds promise for innovative HF therapies. Careful BCAAs supplementation is vital to avoid exacerbating the condition.

BCAAs and their metabolites are also emerging as potential biomarkers for assessing cardiovascular risk, offering insights into disease prediction and prognosis. Further research is needed to explore their effectiveness in predicting a wider range of cardiovascular diseases, such as atherosclerosis, hypertension, and arrhythmias.

## Author contributions

CG: Writing – original draft, Writing – review & editing. LH: Conceptualization, Writing – original draft, Writing – review & editing.
